# Tailored CVD graphene coating as a transparent and flexible gas barrier

**DOI:** 10.1038/srep24143

**Published:** 2016-04-11

**Authors:** Tae Hoon Seo, Seula Lee, Hyunjin Cho, S. Chandramohan, Eun-Kyung Suh, Heon Sang Lee, Su Kang Bae, Soo Min Kim, Min Park, Jae Kwan Lee, Myung Jong Kim

**Affiliations:** 1 Soft Innovative Materials Research Center, Korea Institute of Science and Technology, Jeonbuk 565-905, Republic of Korea; 2 Department of Carbon materials, Chonsun University, Gwangju 501-759, Republic of Korea; 3 School of Semiconductor and Chemical Engineering, Semiconductor Physics Research Center, Chonbuk National University, Jeonju 561-756, Republic of Korea; 4 Department of Chemical Engineering, Dong-A University, Nakdong-Daero 550, Saha-gu, Busan 604-714, Republic of Korea; 5 Photoelectronic Hybrid Research Center, Korea Institute of Science and Technology, Seoul 136-791, Republic of Korea

## Abstract

The chemical vapor deposition (CVD) method to obtain tailored graphene as a transparent and flexible gas barrier has been developed. By separating nucleation step from growth, we could reduce early graphene nucleation density and thus induce better stitching between domain boundaries in the second growth step. Furthermore, two step growth in conjunction with electrochemical polishing of Cu foils achieved large graphene domains and improved graphene quality with minimized defects. The performance of resulting graphene as a gas barrier was superior to the graphene obtained by one-step growth on polished or unpolished Cu foils. The CVD graphene reported here could open up the possibility for exploring graphene-based gas barrier due to the minimized density of defect area.

Graphene, a single atom thick sheet of sp^2^ hybridized carbon atoms arranged in hexagonal lattice, has gained significant interest due to its outstanding physical properties such as mechanical stiffness, very high electrical and thermal conductivity, elasticity, and so on^1^,^2^,^3^,^4^,^5^. Accordingly, much efforts have been dedicated to the fundamental study and various application of graphene-based materials in electronics, energy generation and storage, and biology^1^,^2^,^3^,^4^,^5^,^6^,^7^,^8^. In particular, graphene with chemical inertness and complete impermeability to any gases has been exploited as a gas barrier against water and oxygen diffusion and protective layer to prevent the metal electrodes from oxidation, corrosion, and degradation in electrochemical systems^9^,^10^,^11^,^12^,^13^,^14^,^15^,^16^,^17^.

For practical applications as protective barrier, preparation of large area, defect-free graphene and a suitable method for its integration on polymeric or other substrates are important requirements. Among many preparation methods, chemical vapor deposition (CVD) is the most effective method to reliably produce high-quality, wafer-scale monolayer graphene films. By virtue of this, CVD-grown graphene has been investigated as a protective barrier to prevent transport of gas molecular between the environment and the arbitrary surface such as polymers or metal layer^9^,^10^,^11^,^12^,^13^,^14^,^15^,^16^,^17^. This is a promising step towards exploring alternative materials, because conventionally used organic polymers for barrier application has limited potential due to their relatively high gas permeability as compared to high-criteria demands for modern packaging application and current display devices such as organic light emitting diodes and liquid crystal displays. However, despite the promising properties, CVD graphene layer as a protective gas barrier has been found to result reduced oxidation and corrosion resistance in protecting the metal surfaces^12^,^15^,l^8^,^19^. Also, a recent study showed that the corrosion rate of Cu is accelerated by graphene overlayer compared to bare Cu under exposure for long period^20^. This is because line defects and grain boundaries formed at the interfaces between two domains with different crystallographic orientations of polycrystalline Cu foil allow the transfer of gaseous molecules toward arbitrary surface covered by such graphene layer^15^. Furthermore, CVD graphene contains many defects, *viz*, point defects, wrinkles, folds, tears and cracks, due to the local variations in the properties of the Cu substrate such as its thickness, surface roughness, grain size and orientation, surface impurities, and transfer process. As a consequence, unfavorable gas and aerosol molecules can be transferred through these defects, causing local corrosive change in the surface and degradation of the protection ability of the graphene^18^,^19^. Thus, for barrier applications, it is very important to ensure complete surface coverage by minimizing any grain boundaries or structural defects. Numerous approaches have been developed to reduce the nucleation density and point defects as well as to increase the graphene domain size^21^,^22^,^23^,^24^,^25^,^26^,^27^,^28^,^29^. Alternatively, it has been demonstrated that the corrosion rate of metallic surfaces can be reduced by controlling the number of graphene layers and multilayer graphitic films formed by gentle chemical reduction of graphene oxide laminates with hydroiodic and ascorbic acids were found to furnish high-quality barrier films that obstruct all gases and liquids^12^,^16^. Hsieh *et al*.^15^ introduced a method to selectively passivate the defects in graphene by using atomic layer deposition (ALD) of aluminum oxide, which offered enhanced corrosion protection to graphene. However, the above approaches have some disadvantages such that an increment in the number of graphene layers diminishes the transmittance and an additional transfer process reduces the production yields. Also, the ALD is very expensive and hence its broad application is still limited because of a lack of scale-up.

Here, we use a combined approach of electro-chemical polishing (ECP) and two-step growth methods to improve the gas barrier property of graphene without an increase in the layer numbers or additional fabrication process. ECP is known as a highly effective step to reduce the surface roughness of Cu foil and remove the impurity particles^30^. Both effects could decrease the point defects and wrinkles within the graphene layer and increase the graphene domain size. However, single-crystal terraces and step edges unintentionally exist on Cu foils during graphene growth in the CH_4_/H_2_ mixture which act as nucleation seeds and can influence the gas barrier properties of graphene^26^,^31^. In particular, the two-step growth approach reported here is facile and simple. From the understating that the nucleation density is governed by carbon supply rate, a nucleation step was decoupled from the growth to achieve high quality graphene. By keeping the carbon supply rate low during the initial stage of graphene growth, a low nucleation density is achieved and a successive fast lateral growth is dictated by a high carbon supply rate to achieve continuous graphene. The simple two-step growth approach led to better stitching between domain boundaries.

## Results

**Figure 1:**
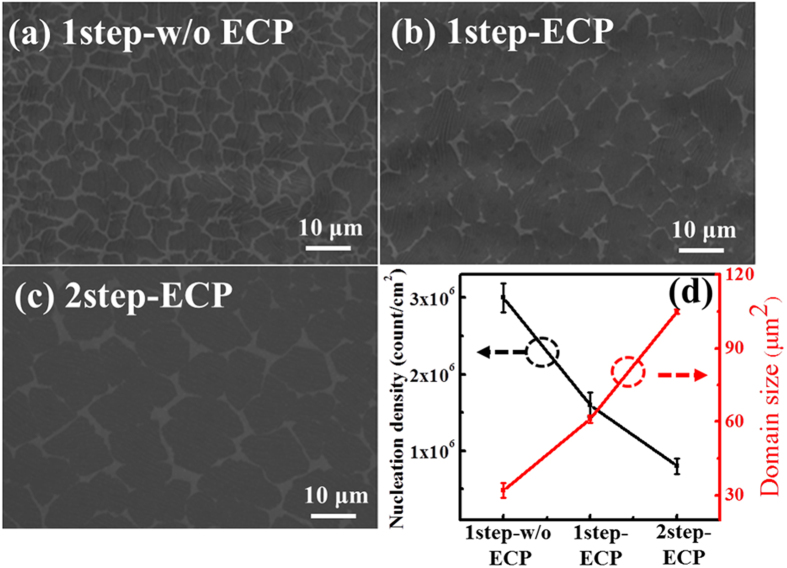
SEM images of graphene on copper at the initial stage of growth after 1 min. (**a**) 1step-w/o ECP, (**b**) 1step-ECP and (**c**) 2step-ECP. (**d**) Variations in nucleation density and domain size for three differently grown graphene.

The initial stage of growth is critical to obtain large size domains and high quality graphene layer. In this study, three different graphene layers are grown as follows: The first graphene was grown on as-received Cu foil without any treatment by one step growth (1step-w/o ECP), the second graphene was grown on electrochemically polished Cu foil by one step growth (1step-ECP) and the third graphene was grown on ECP Cu foil by two step growth (2step-ECP). Figure 1(a–c) show the scanning electron microscopy (SEM) images of the three different graphene samples at early stage of growth after 60 sec, respectively. All graphene domains on either substrates show irregular shape, similar to previously reported graphene domains grown by CVD^21^,^22^,^23^. According to previous studies on the CVD growth of graphene, the CH_4_ to H_2_ flow ratio, total pressure, temperature, and Cu foil treatments are crucial parameters that influence the graphene domain shape^21^,^22^,^23^,^24^,^25^,^26^. Interestingly, the two-step growth on ECP Cu foil resulted in large graphene domains with an average size of approximately 110 μm^2^, twice or four times larger than the graphene domains obtained by one-step growth on ECP and unpolished Cu foils, respectively, as illustrated in Fig. 1(d). Furthermore, it is observed that the increase in domain size results in a decrease in the nucleation density, which is estimated to be 0.7 × 10^6^/cm^2^ in the case of two-step growth on ECP Cu. It is well known that steps, wrinkles, defects, and grain boundaries in Cu foils have much lower nucleation barriers than flat surface. These could act as nucleation sites, triggering the formation of graphene nuclei and eventually give rise to small single-crystal graphene domains. On the other hand, ECP process reduces the surface roughness as well as defects and impurities that subsist on Cu foils, leading to the suppression of nucleation sites and hence reduced domain density of graphene. In addition, during the early stage of two-step growth, the low CH_4_ concentration also mitigates the graphene nucleation and provides space for the growth of large graphene domains. As a result, two-step growth of graphene on ECP Cu foil gives large-size single crystalline graphene domains with a reduced domain density.

**Figure 2:**
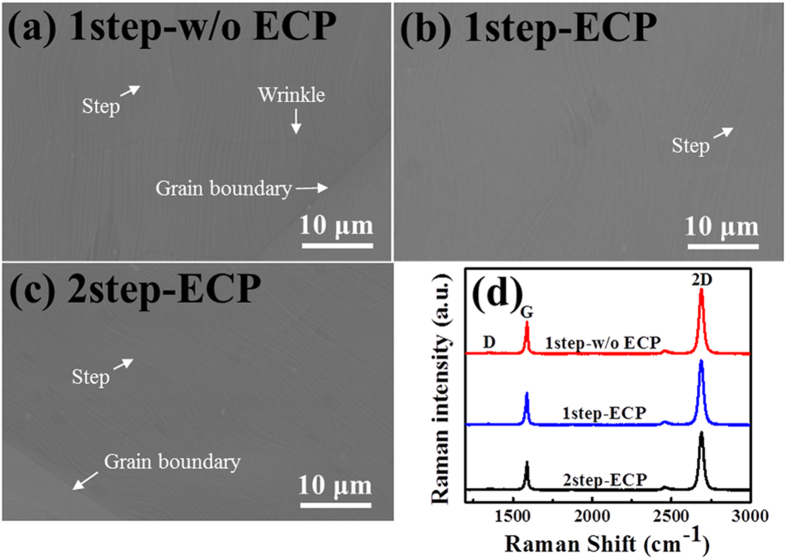
(**a**–**c**) SEM images and (**d**) Raman spectra of fully covered 1step-w/o ECP, 1step-ECP, and 2step-ECP graphene films.

Figure 2(a–c) show the SEM images of the three different graphene films on Cu foils. One can notice that all the three graphene films are continuous, uniform and clean without noticeable particles, but grain boundaries, Cu surface steps, and wrinkles are observed, which could influence the gas barrier property of graphene. This will be addressed further later with water vapor transmission rate (WVTR) results.

To investigate the quality and number of graphene layers, Raman measurements were performed on representative graphene films after being transferred on to SiO_2_/Si substrate. Figure 2(d) compares the Raman spectra of the three different graphene films. Two predominant peaks i.e., the G-band and the 2D-band associated with graphene are observed from all the samples at 1589 cm^−1^ and 2690 cm^−1^, respectively. However, the G-band position is found to be blue shifted compared to the value 1582 cm^−1^ reported in the literature^32^. This is due to unintentional charge transfer induced *p*-type doping of graphene caused by the redox reaction between oxygen from air and adsorbed water at the surface of SiO_2_/Si substrate having hydrophilic property^33^. Raman spectra obtained from all samples exhibit a feature of monolayer graphene, i.e. a single Lorentzian peak with a full width at half maximum (FWHM) of 30 cm^−1^ and the intensity ratio of 2D to G greater than 2. Also, the so-called defects or disorder-induced D-band peak is seen around 1350 cm^−1^ at about half of the frequency of the 2D band with negligible intensity.

**Figure 3:**
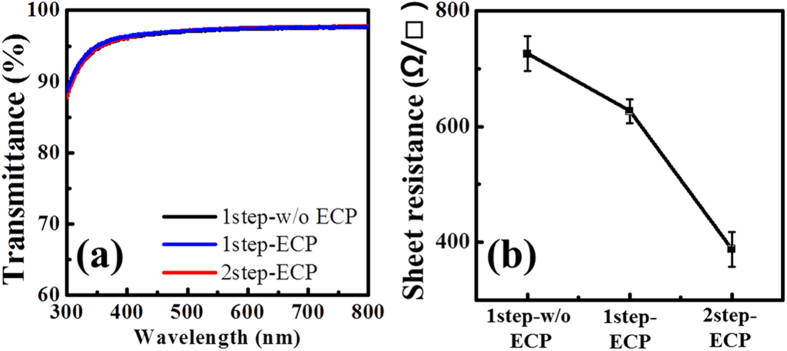
(**a**) Optical transmittance characteristics and (**b**) sheet resistance of three differently grown graphene.

Figure 3(a,b) show the opto-electrical properties of the three graphene films under study. In general, monolayer graphene absorbs nearly 2.3% of the incident light at the wavelengths over 500 nm^34^. As shown in Fig. 3(a), the transmittance of all graphene samples transferred on to glass substrate is about 97.4% at the wavelength of 550 nm, which deviates slightly from the expected value of 97.7%. However, this is a general trend since monolayer graphene grown on Cu foils by CVD contains some portion of bilayer, depending on growth conditions.

Even though the transmittance of 2step-ECP graphene films shows no difference compared with that of 1step-w/o ECP or the 1step-ECP graphene films, sheet resistance change reflects graphene quality rather than thickness. In Fig. 3(b), the sheet resistance of 2step-ECP graphene films is estimated to be 388 ± 20 Ω/□, a value lower than the values of 726 ± 30 Ω/□ and 627 ± 20 Ω/□ measured for the 1step-w/o ECP and the 1step-ECP graphene films, respectively. The observed low sheet resistance can be understood as follows: Line defects and grain boundaries formed at the interfaces between two domains with different crystallographic orientations of polycrystalline Cu foil can scatter charge carriers and disturb their transport toward lateral direction. This suppresses the ballistic transport path length and leads to high sheet resistance for the graphene films with a higher domain density. By increasing the domain size, the influence of grain boundaries can be reduced because the probability for carriers encountering the grain boundaries decreases, which as a consequence could reduce the sheet resistance of graphene. Since the ECP-2step graphene had relatively large domain size with reduced domain density, its sheet resistance decreased remarkably. Previous studies also demonstrated similar enhancement in the electrical conductivity of graphene as a result of Cu pretreatment and growth conditions^30^,^35^,^36^ and the lowest sheet resistance reported to date is around 210 Ω/□ for a CVD graphene on electroplolished Cu^30^, while the reported best value is still around 125 Ω/□ for a 30 inch graphene produced by CVD^35^. The relatively higher sheet resistance of our graphene could be likely due to difference in growth conditions, the transfer, and measurement conditions.

**Figure 4:**
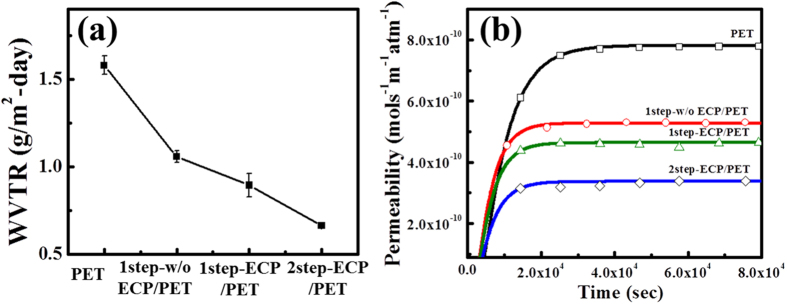
(**a**) WVTR and (**b**) water vapor permeability (P) of various graphene samples studied in this work.

Figure 4(a,b) show WVTR results and water permeability with time for three different graphene samples. All the three samples reached a steady-state of WVTR within 24 hours. Corresponding data for pure poly ethylene terephthalate (PET) is also shown for comparison. As can be seen in Fig. 4(a) that the formation of graphene on PET leads to a decrease WVTR compared to bare PET, indicating that graphene acts as a protecting layer for PET against water owing to its impermeable property. In particular, 2step-ECP graphene on PET exhibits the lowest WVTR value of 0.665 ± 0.046 g/m^2^-day, which is 57.9% less than that of the value of 1.58 ± 0.029 g/m^2^-day measured for the bare PET. Figure 4(b) depicts the WVTR fitting results through the Fick’s second law of diffusion^37^. The water vapor permeability (P) values for the 1step-w/o ECP, 1step-ECP, and 2step-ECP graphene films on PET are found to be 6.608 × 10^−14^, 5.806 × 10^−14^ and 4.224 × 10^−14^ mols^−1^ m^−1^atm^−1^, respectively. It is evident that the lowest WVTR and P values are obtained for monolayer graphene formed by two-step growth on ECP Cu foil without any additional treatment or control of graphene number.

**Figure 5:**
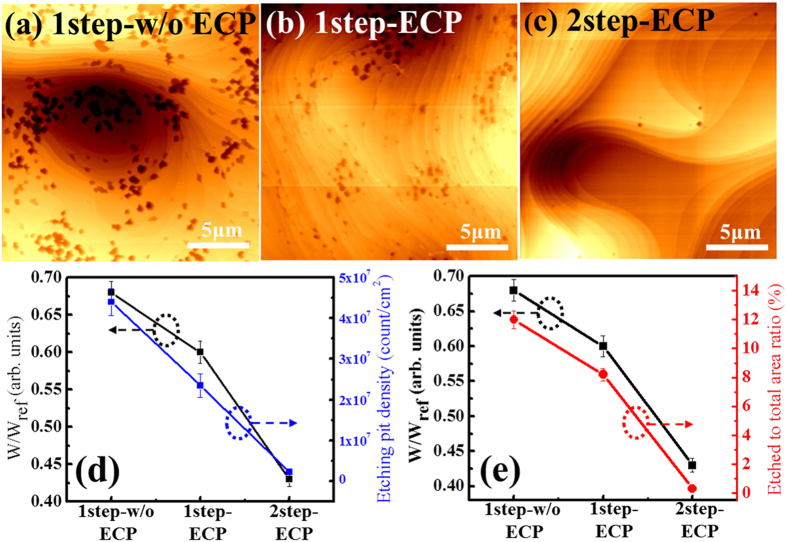
(**a**–**c**) AFM images of the surface of graphene films on native Cu substrate after dropping and washing copper etchant. (**d**,**e**) Correlation between the ratio of WVTR of graphene to WVTR of PET and the etching pit density or etched to the total area ratio (Δ).

Though Raman analysis can well assess the defect distribution and quality of graphene through the G-to-D band intensity ratio, it is hard to measure the spatial distribution of defects that spread across over large area. To give a further insight into the structural quality of graphene on large scale, a film-induced frustrated etching (FIFE) is carried out, which is a simple and facile method to observe the structural defects spread on large area of graphene^38^. This method allows us to easily analyze the differences in the sample quality. To perform this study, a commercial copper etchant, ammonium persulfate [(NH_4_)_2_S_2_O_8_], is dropped onto graphene synthesized on Cu foils. After waiting for about 5 sec, the dropped etchant is removed through rinsing process with deionized water and subsequently the surface is examined by atomic force microscope (AFM), as shown in Fig. 5(a–c). It is expected that etching of copper occurs in regions that are not covered by graphene, which include partially grown areas, lattice or line defects, and particles on the Cu surface. The etch pit densities for 1step-w/o ECP, 1step-ECP, and 2step-ECP graphene are found to be 4.4 × 10^−7^ ± 3.3 × 10^−6^/cm^2^, 2.3 × 10^−7^ ± 2.8 × 10^−6^/cm^2^, and 2.2 × 10^−6^ ± 6.0 × 10^−5^/cm^2^, respectively. It is evident that the graphene grown by two-step method on ECP Cu has the lowest etch pit density, signifying that the two-step growth method reduces the defect density to a great extent compared to conventional one-step growth. Figure 5(d,e) show the correlation between ratio of graphene/PET WVTR (W) to bare PET WVTR (W_PET_) and etch pit density and etched to the total area ratio (Δ) obtained by a commercial image processor (ImageJ 1.42q, NIH) from corresponding AFM images. The Etch pit density shows a linear decrease from the first sample (1step-w/o ECP) to the third one (2step-ECP), which is in accordance with the SEM results shown in Fig. S5. Moreover, Δ of graphene obtained by one-step growth is found to decrease when the Cu foil is subjected to ECP. A further considerable decrease in Δ is obvious for graphene grown by two-step method on ECP Cu foil, as shown in Fig. 5(e). This result signifies that the enhanced barrier property of graphene stems largely from the two-step growth process rather than electrochemical polishing. It should be noted that Δ curve is similar to that of WVTR results, indicating that barrier performance of graphene actually corresponds to the etched area than etched defect density.

## Discussion

**Figure 6:**
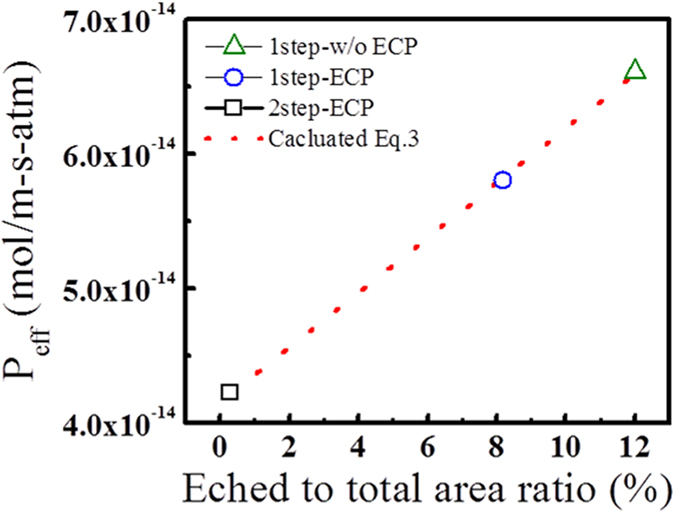
The water vapor permeability of the three different graphene layer/PET film as a function of Δ given in Fig. 5. The symbols are experimental data and the dotted line is a fit with by Eq. (3).

The solution of the Fick’s second law of diffusion is used to fit the measured WVTR, by assuming the water vapor concentration is close to zero at the exit side of the film as given below^37^.


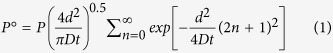


where *P*^*0*^ = *Jd, P* = *J*_*s*_*d* = *SD, J* and *J*_*s*_ are the water vapor molar flux at time t and at steady state, *d* is the sample thickness, *P* is permeability, *D* is diffusivity, and *S* is solubility. If the whole area of PET film is fully covered by impermeable graphene layer, the permeability should be zero. However, the CVD graphene layer consists of defect-holes through which water molecules may diffuse. The water molecules in the PET film may diffuse out through the defect-holes. The surface defect-hole density, ψ, can be defined as the ratio between the defect-hole area and the total area. Inside of the defect-holes, the inhibition of water diffusion by graphene layer may be negligible, when the sizes of the holes are much larger than those of the water molecules and the thickness of hole is much smaller than the mean free path of water molecules. In this case, the permeability can be simply expressed as


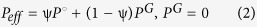


because graphene without defects can be assumed as an impermeable membrane in the simple model where *P*_*eff* _, *P*^*G*^ and *P*° represent the permeability of graphene layer/PET film, bare graphene film, and pure PET film, respectively. However, *P*^*G*^ is not zero but a tiny value due to undetected defects, and thus the equation can be approximated in the form.


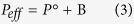


where *B* is a correction constant arising from undetected defects by the FIFE. It is reasonable to assume that the Δ is proportional to ψ such as ψ = AΔ. Then the reduced permeability can be related to Δ as follows;


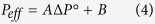


where A is an arbitrary constant. The permeability of graphenes on PET with Δ examined in Fig. 5 is shown in Fig. 6, wherein model calculations by Eq. (3) are used to fit the experimental data. From this result, A and B are estimated to be 2.080 ± 0.027 and 4.157 × 10^−14^ ± 2.205 × 10^−16^, respectively. Thus, the ψ value of graphene grown by 2step-ECP on PET is found to be 0.624 ± 0.0081. This result indicates that the formation of graphene layer on PET is an effective way to improve the water vapor barrier property of the polymer film. If barrier performances of graphene are further improved by optimizing growth conditions, it can be used as transparent and flexible gas barrier in display devices.

In conclusion, a combination of electrochemical polishing and two-step growth is demonstrated to obtain high-quality monolayer graphene by low pressure CVD. This method allows achieving large size graphene domains by controlling the nucleation density of graphene on Cu foils, a crucial starting step for the realization of high-quality graphene. The characteristics of the graphene were compared to that of graphene produced by one-step growth on both unpolished and electrochemically polished Cu foils. Two-step growth on electrochemically polished Cu also led to improved permeability property. This is attributed to the decrease in structural defects and suppression of nucleation of graphene at the initial stage. A simple and accurate model has been proposed to examine the barrier property of CVD monolayer graphene. Our study also brings into picture that the effective permeability of CVD monolayer graphene is proportional to defect area rather than defect density.

## Methods

### Electro-chemical polishing (ECP)

ECP was performed in diluted 3H_3_PO_4_:H_2_O acid solution at a constant voltage of 2.0 V for 12 min using Cu plate as cathode and Cu foil (80 mm × 80 mm) as working electrode.

### Graphene synthesis

Large-scale graphene layer studied in this work was synthesized on 35-μm-thick Cu foils (Nippon Mining) by low pressure chemical vapor deposition (LPCVD). The Cu foil was then set into a 2 inch quartz tube and heated by split-tube furnace. Meanwhile, quartz tube was pumped down to 0.072 Torr, and H_2_ gas was flown through the reactor at 15 standard cubic centimeters per minute (sccm) during the temperature ramp-up up to 1030 °C. Then, the Cu foils were annealed for 50 min. In the one-step growth, CH_4_ of 13 sccm and H_2_ of 15 sccm were supplied at 1030 °C for 23 min under a growth pressure of 0.160 Torr. In the case of two-step growth, at first, large size graphene domains were formed on Cu foil in a mixed CH_4_/H_2_ (5 sccm/100 sccm) ambient for 1 min at 1030 °C under a growth pressure of 0.365 Torr. This is the optimal condition for obtaining uniform and large domains of graphene, as shown in the Figure S3 of supporting information. The growth of continuous graphene was then achieved during the second growth step when the flow rate of CH_4_ and growth time were increased to 13 sccm and 8 min, respectively, while maintaining the H_2_ flow rate and temperature unchanged. Finally, the samples were cooled down rapidly to room temperature with a cooling rate of 30 °C min^−1^ at 15 sccm of H_2_ flow by moving the furnace out of the substrate region.

### Graphene transfer

Polymethyl methacrylate (PMMA) was spin-coated onto graphene surface at 4200 rpm for 50 s to protect and transfer the graphene layer onto other substrates of interest. Prior to graphene transfer, graphene unintentionally formed on the back side of the Cu foil was removed by O_2_ plasma etching. Then, the Cu foil with PMMA-covered graphene was dipped in 0.1 M Ammonium Persulfate [(NH_4_)_2_S_2_O_8_] solution for 4 h to etch the Cu foil. Thereafter, PMMA/graphene layer was transferred onto various substrates, such as 300 nm SiO_2_/Si substrate, PET film, and glass, to investigate the opto-electrical and gas barrier properties. The PMMA was removed by using acetone and the sample was annealed in an Ar/H_2_ gas mixture at 400 °C for 3 h to eradicate remained PMMA residues.

### Film-induced frustrated etching (FIFE)

Commercial copper etchant, ammonium persulfate [(NH_4_)_2_S_2_O_8_], was dropped on to graphene synthesized on Cu foils. After 5 sec, the dropped etchant was removed by rinsing with deionized water.

### Water vapor transmission rate (WVTR) measurement

WVTR measurements were performed in a commercial MOCON’s proprietary AQUATRAN model 2 equipment at 100% relative humidity and room temperature under 1 atm. Water vapor was filled on the upper surface of the sample and the bottom surface of the sample was filled by a nitrogen gas. Molecules of water diffuse via the sample to the bottom surface and are detected to a gold sensor by the nitrogen gas.

### Characterization

Field emission scanning electron microscopy (FESEM, NovaSEM 450) was used to evaluate the surface morphology of the graphene samples studied in this work. The surface topography of graphene/Cu foils after FIFE test was probed by atomic force microscope (AFM, Park NX10) in tapping mode. The Raman spectra were recorded using 514 nm-line of an Ar ion laser as an excitation source. In order to observe the change in electrical conductivity as a function of graphene domain size, the sheet resistance was measured by Van der Pauw method using Hall measurement system (Lakeshore 7500 serises).

## Additional Information

**How to cite this article**: Seo, T. H. *et al*. Tailored CVD graphene coating as a transparent and flexible gas barrier. *Sci. Rep.* **6**, 24143; doi: 10.1038/srep24143 (2016).

## Supplementary Material

Supplementary Information

## References

[b1] Novoselov K. S. . Electric Field Effect in Atomically Thin Carbon Films. Science 306, 666–669 (2004). 15499015 10.1126/science.1102896

[b2] Zhang Y., Tan Y. W., Stormer H. L. & Kim P. Experimental observation of the quantum Hall effect and Berry’s phase in graphene. Nature 438, 201–204 (2005). 16281031 10.1038/nature04235

[b3] Lee C., Wei X., Kysar J. W. & Hone J. Measurement of the Elastic Properties and Intrinsic Strength of Monolayer Graphene. Science 321, 385–388 (2008). 18635798 10.1126/science.1157996

[b4] Morozov S. V. . Giant Intrinsic Carrier Mobilities in Graphene and Its Bilayer. Phys. Rev. Lett. 100, 016602 (2008). 18232798 10.1103/PhysRevLett.100.016602

[b5] Balandin A. A. Thermal properties of graphene and nanostructured carbon materials. Nature Mater 10, 569–581 (2011). 21778997 10.1038/nmat3064

[b6] Novoselov K. S. . A roadmap for graphene. Nature 490, 192–200 (2012). 23060189 10.1038/nature11458

[b7] Zhu Y. . Graphene and Graphene Oxide: Synthesis, Properties, and Applications. Adv. Mater 22, 3906–3924 (2010). 20706983 10.1002/adma.201001068

[b8] Huang X. . Graphene-Based Electrodes. Adv. Mater 24, 5979–6004 (2012). 22927209 10.1002/adma.201201587

[b9] Bunch J. S. . Impermeable Atomic Membranes from Graphene Sheets. Nano Lett. 8, 2458–2462 (2008). 18630972 10.1021/nl801457b

[b10] Chen S. . Oxidation Resistance of Graphene-Coated Cu and Cu/Ni Alloy. ACS Nano 5, 1321–1327 (2011). 21275384 10.1021/nn103028d

[b11] Kirkland N. T., Schiller T., Medhekar N. & Birbilis N. Exploring graphene as a corrosion protection barrier. Corros. Sci. 56, 1–4 (2012).

[b12] Prasai D. . Graphene: Corrosion-Inhibiting Coating. ACS Nano 6, 1102–1108 (2012). 22299572 10.1021/nn203507y

[b13] Raman R. K. S. . Protecting copper from electrochemical degradation by graphene coating. Carbon 50, 4040–4045 (2012).

[b14] Berry V. Impermeability of graphene and its applications. Carbon 62, 1–10 (2013).

[b15] Hsieh Y.-P. . Complete Corrosion Inhibition through Graphene Defect Passivation. ACS Nano 8, 443–448 (2014). 24359599 10.1021/nn404756q

[b16] Su Y. . Impermeable barrier films and protective coatings based on reduced graphene oxide. Nature Commun 5, 4843 (2014). 25208890 10.1038/ncomms5843

[b17] Choi K. . Reduced Water Vapor Transmission Rate of Graphene Gas Barrier Films for Flexible Organic Field-Effect Transistors. ACS Nano 9, 5818–5824 (2015). 25988910 10.1021/acsnano.5b01161

[b18] Wlasny I. . Role of graphene defects in corrosion of graphene-coated Cu(111) surface. Appl. Phys. Lett. 102, 111601 (2013).

[b19] Zhang Y. H. . Role of wrinkles in the corrosion of graphene domain-coated Cu surfaces. Appl. Phys. Lett. 104, 143110 (2014).

[b20] Schriver M. . Graphene as a Long-Term Metal Oxidation Barrier: Worse Than Nothing. ACS Nano 7, 5763–5768 (2013). 23755733 10.1021/nn4014356

[b21] Li X. . Graphene Films with Large Domain Size by a Two-Step Chemical Vapor Deposition Process. Nano lett. 10, 4328–4334 (2010). 20957985 10.1021/nl101629g

[b22] Vlassiouk I. . Role of Hydrogen in Chemical Vapor Deposition Growth of Large Single-Crystal Graphene. ACS Nano 5, 6069–6076 (2011). 21707037 10.1021/nn201978y

[b23] Zhang Y. . Vapor Trapping Growth of Single-Crystalline Graphene Flowers: Synthesis, Morphology, and Electronic Properties. Nano lett. 12, 2810–2816 (2012). 22536825 10.1021/nl300039a

[b24] Wang H. . Controllable Synthesis of Submillimeter Single-Crystal Monolayer Graphene Domains on Copper Foils by Suppressing Nucleation. J. Am. Chem. Soc. 134, 3627–3630 (2012). 22324740 10.1021/ja2105976

[b25] Regmi M., Chisholm M. F. & Eres G. The effect of growth parameters on the intrinsic properties of large-area single layer graphene grown by chemical vapor deposition on Cu. Carbon 50, 134–141 (2012).

[b26] Kraus J. . Towards the perfect graphene membrane? – Improvement and limits during formation of high quality graphene grown on Cu-foils. Carbon 64, 377–390 (2013).

[b27] Vlassiouk I. . Large scale atmospheric pressure chemical vapor deposition of graphene. Carbon 54, 58–67 (2013).

[b28] Vlassiouk I. . Graphene Nucleation Density on Copper: Fundamental Role of Background Pressure. J. Phys. Chem. C 117, 18919–18926 (2013).

[b29] Kraus J., Böbel M. & Günthe S. Suppressing graphene nucleation during CVD on polycrystalline Cu by controlling the carbon content of the support foils. Carbon 96, 153–165 (2016).

[b30] Lee D. . Significant enhancement of the electrical transport properties of graphene films by controlling the surface roughness of Cu foils before and during chemical vapor deposition. Nanoscale 6, 12943–12951 (2014). 25233143 10.1039/c4nr03633f

[b31] Ni G.-X. . Quasi-Periodic Nanoripples in Graphene Grown by Chemical Vapor Deposition and Its Impact on Charge Transport. ACS nano 6, 1158–1164 (2012). 22251076 10.1021/nn203775x

[b32] Malard L. M., Pimenta M. A., Dresselhaus G. & Dresselhaus M. S. Raman spectroscopy in graphene. Phys. Rep. 473, 51–87 (2009).

[b33] Levesque P. L. . Probing Charge Transfer at Surfaces Using Graphene Transistors. Nano Lett. 11, 132–137 (2011). 21141990 10.1021/nl103015w

[b34] Nair R. R. . Fine Structure Constant Defines Visual Transparency of Graphene. Science 320, 1308 (2008). 18388259 10.1126/science.1156965

[b35] Bae S. . Roll-to-roll Production of 30-inch Graphene Films for Transparent Electrodes. Nature Nanotechnology 5, 574–578 (2010). 10.1038/nnano.2010.132 20562870

[b36] Yan J. . Toward the Synthesis of Wafer-Scale Single-Crystal Graphene on Copper Foils. ACS Nano 6, 9110–9117 (2012). 22966902 10.1021/nn303352k

[b37] Kim H. M., Lee J. K. & Lee H. S. Transparent and high gas barrier films based on poly(vinyl alcohol)/graphene oxide composites. Thin Solid Films 519, 7766–7771 (2011).

[b38] Hofmann M. . A Facile Tool for the Characterization of Two-Dimensional Materials Grown by Chemical Vapor Deposition. Nano Res. 5, 504–511 (2012).

