# Supplementary material for: Tailored CVD graphene coating as a transparent and flexible gas barrier
Source: Sci Rep. 2016 Apr 11;6:24143. doi: 10.1038/srep24143 (PMC4827024; doi:10.1038/srep24143)
Supplement: Supplementary Information [file srep24143-s1.pdf]

Tailored CVD graphene coatings as a transparent and flexible gas barrier

Tae Hoon Seo,[1] Seula Lee,[1,4] Hyunjin Cho,[1] S. Chandramohan,[3] Eun-Kyung Suh,[3] Heon Sang Lee,[5] Su Kang Bae,[1] Soo Min Kim,[1] Min Park,[2] Jae Kwan Lee,[4] and Myung Jong Kim [1,]*

[1] Soft Innovative Materials Research Center and [2] Photoelectronic Hybrid Research Center, Korea Institute of Science and Technology, Jeonbuk 565-905[1] and Seoul 136-791[2], Republic of Korea

[3] School of Semiconductor and Chemical Engineering, Semiconductor Physics Research Center, Chonbuk National University, Jeonju 561-756, Republic of Korea

[4] Department of Carbon materials, Chonsun University, Gwangju 501-759, Republic of Korea

[5] Department of Chemical Engineering, Dong-A University, Nakdong-Daero 550, Saha-gu, Busan 604-714, Republic of Korea

---

* **Corresponding author**. Tel.: +82-63-219-8135

E-mail: myung@kist.re.kr (M. J. Kim)

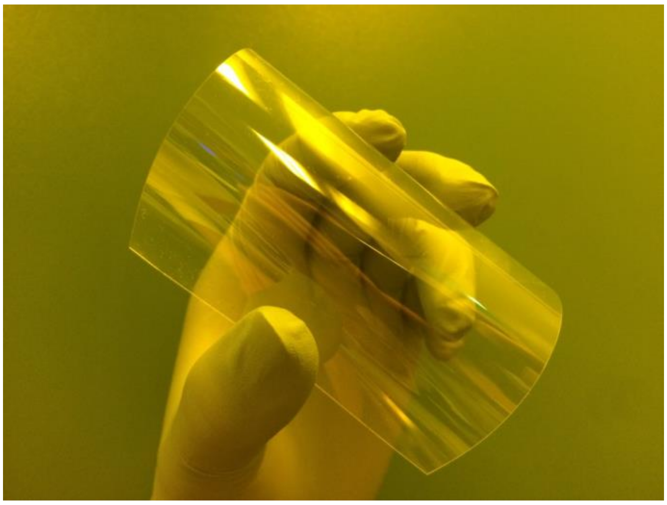

Figure S1. Photograph of graphene transferred on to PET film. The image showing excellent flexibility.

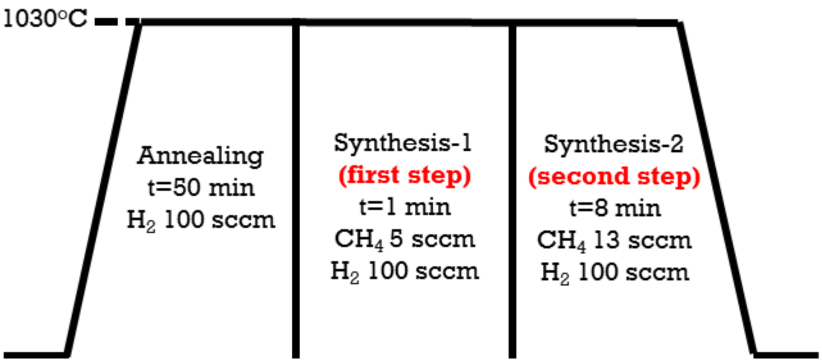

Figure S2. Schematic illustration of two-step growth processes.

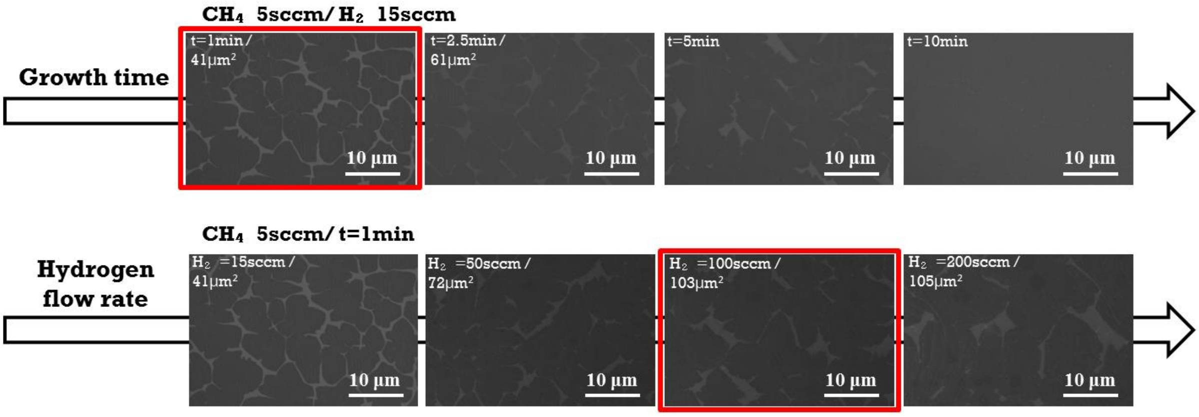

Figure S3. SEM images showing the effect of growth time and hydrogen flow rate at first step. The optimum condition for large domain graphene was 1 min of growth with hydrogen flow rate of 100 sccm.

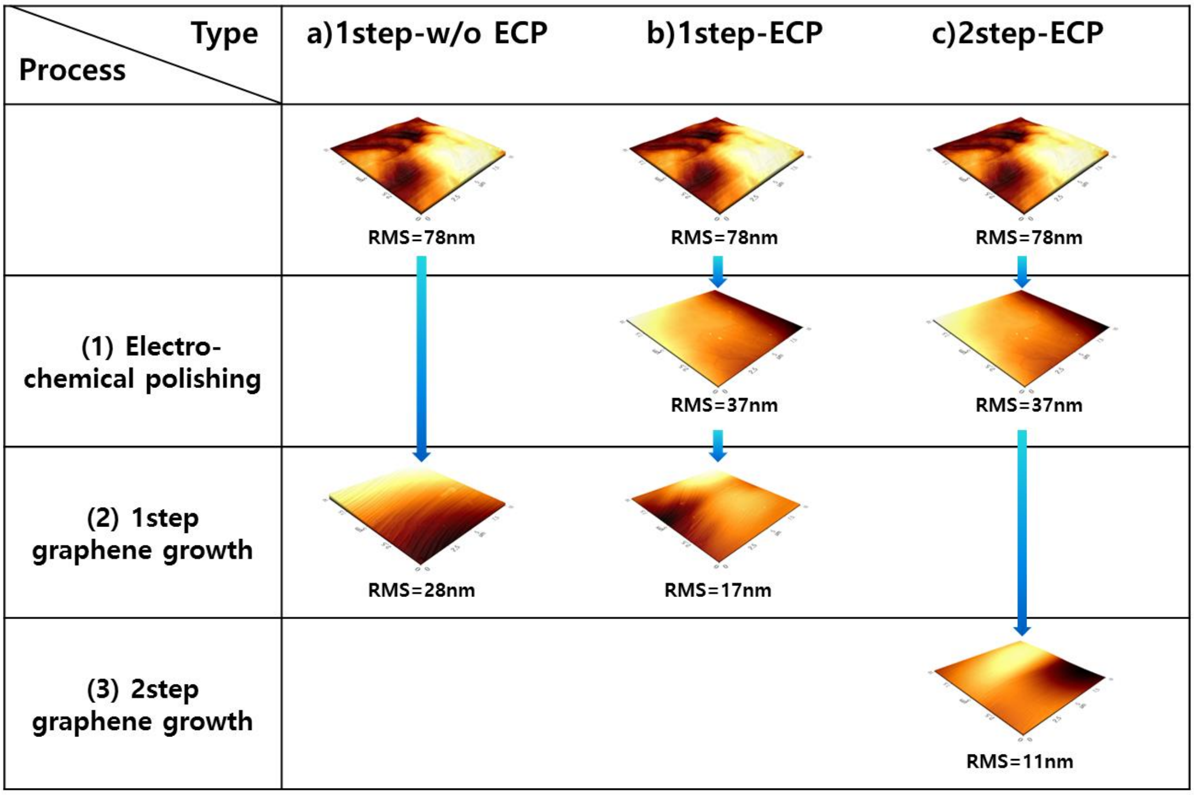

Figure S4. AFM images and root mean square (RMS) roughness values of Cu foils that went through various graphene growth processes. (a) 1step-w/o ECP (b) 1step-ECP and (c) 2step-ECP, respectively. The scan size is 10×10 μm^2.

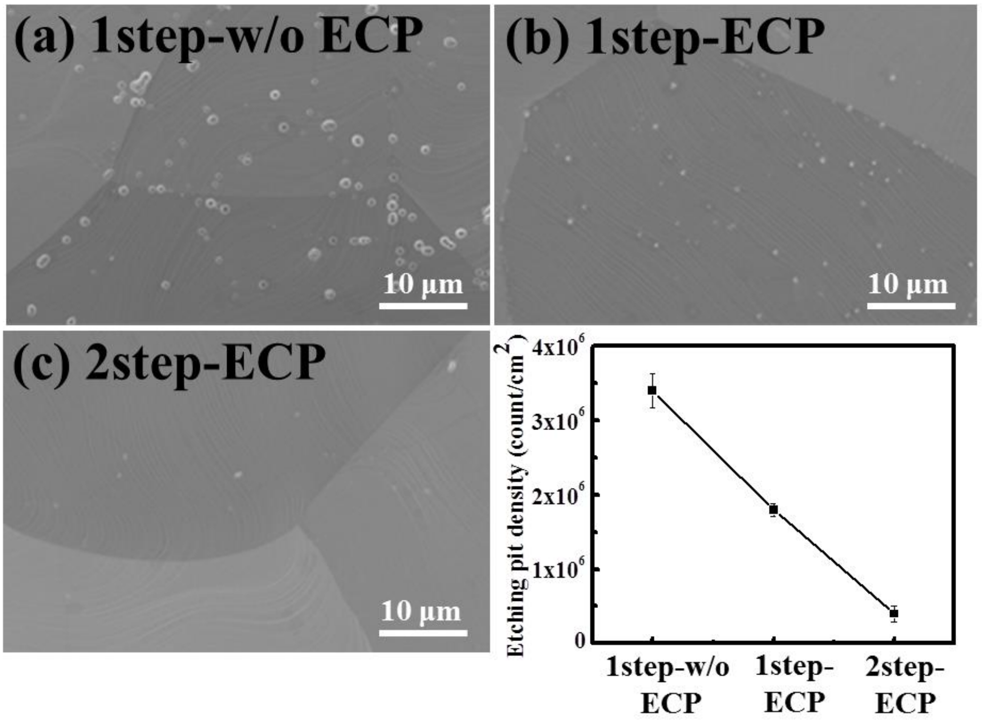

Figure S5. (a)-(c) SEM images of graphene surface on Cu foils after the copper etchant dropping, and (d) their etching pit density. The defect density shows a linear decrease from 1step-w/o ECP to 2step-ECP.

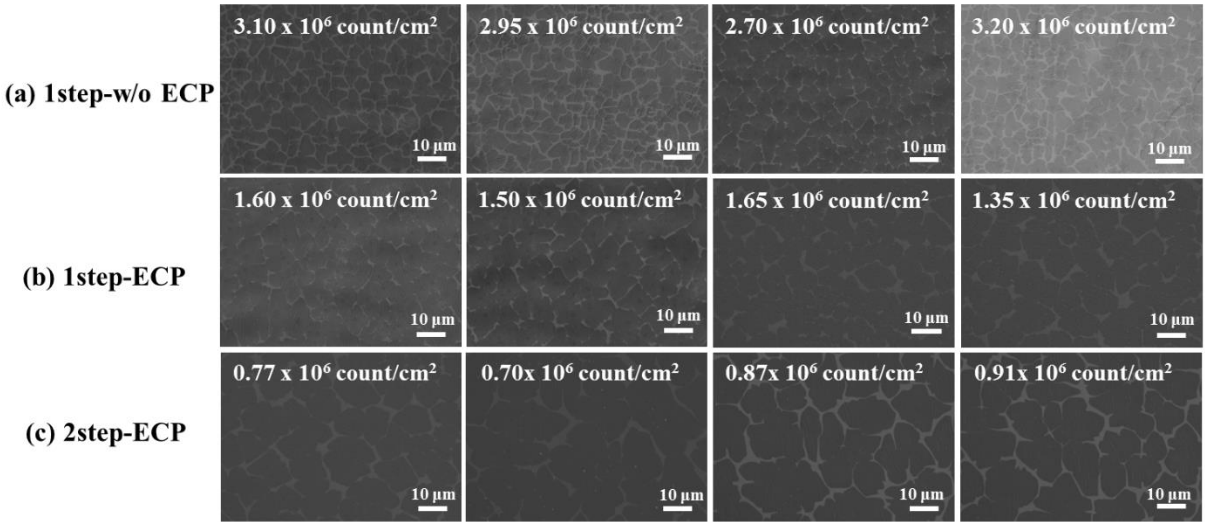

Figure S6. SEM images measured four times in different position of graphenes grown (a) 1step-w/o ECP, (b) 1step-ECP, and (c) 2step-ECP, respectively.

Analysis/ Sample type	Domain size	Raman 2D/G ratio	Transmittance	Sheet resistance	WVTR	Etching pit density
a)1step-w/o ECP	32 μm^2	2.07	97.2757 %	726 Ω/□	1.058 g/m^2-day	$4.25x10^7$ $Count/cm^2$
	28 μm^2	2.03	97.3715 %	702 Ω/□	1.066 g/m^2-day	$4.87x10^7$ $Count/cm^2$
	35 μm^2	2.11	97.3829 %	724 Ω/□	1.023 g/m^2-day	$4.40x10^7$ $Count/cm^2$
	35 μm^2	2.07	97.7334 %	752 Ω/□	1.086 g/m^2-day	$4.71x10^7$ $Count/cm^2$
	30 μm^2	2.00	97.8395 %	693 Ω/□	1.082 g/m^2-day	$4.37x10^7$ $Count/cm^2$
b)1step-ECP	60 μm^2	2.00	97.3829 %	681 Ω/□	0.959 g/m^2-day	$2.00x10^7$ $Count/cm^2$
	58 μm^2	2.16	97.4405 %	634 Ω/□	0.894 g/m^2-day	$2.56x10^7$ $Count/cm^2$
	66 μm^2	2.32	97.3787 %	644 Ω/□	0.899 g/m^2-day	$2.35x10^7$ $Count/cm^2$
	69 μm^2	2.07	97.3684 %	603 Ω/□	0.825 g/m^2-day	$2.31x10^7$ $Count/cm^2$
	52 μm^2	2.19	97.3620 %	633 Ω/□	0.890 g/m^2-day	$2.19x10^7$ $Count/cm^2$
c)2step-ECP	100 μm^2	2.01	97.2752 %	388 Ω/□	0.654 g/m^2-day	$0.25x10^7$ $Count/cm^2$
	120 μm^2	2.23	97.3299 %	351 Ω/□	0.677 g/m^2-day	$0.23x10^7$ $Count/cm^2$
	112 μm^2	2.02	97.8583 %	399 Ω/□	0.664 g/m^2-day	$0.31x10^7$ $Count/cm^2$
	94 μm^2	2.21	97.6690 %	414 Ω/□	0.665 g/m^2-day	$0.18x10^7$ $Count/cm^2$
	99 μm^2	2.15	97.1979 %	355 Ω/□	0.657 g/m^2-day	$0.20x10^7$ $Count/cm^2$

Table S1. The various properties summarized of the three different samples studied in this work.
